# Brain Changes in Long-Term Zen Meditators Using Proton Magnetic Resonance Spectroscopy and Diffusion Tensor Imaging: A Controlled Study

**DOI:** 10.1371/journal.pone.0058476

**Published:** 2013-03-25

**Authors:** Nicolás Fayed, Yolanda Lopez del Hoyo, Eva Andres, Antoni Serrano-Blanco, Juan Bellón, Keyla Aguilar, Ausias Cebolla, Javier Garcia-Campayo

**Affiliations:** 1 Department of Radiology, Hospital Quirón, Zaragoza, Spain; 2 Department of Psychology & Sociology, University of Zaragoza, Zaragoza, Spain; 3 CIBER Epidemiología y Salud Pública, Unidad Epidemiología Clínica, Hospital 12 de Octubre, Madrid, Spain; 4 Parc Sanitari Sant Joan de Déu & Fundació Sant Joan de Déu. Sant Boi de Llobregat, Barcelona, Spain; 5 Centro de Salud El Palo, Unidad de Investigación del Distrito de Atención Primaria de Málaga (redIAPP, grupo SAMSERAP), Departamento de Medicina Preventiva, Universidad de Málaga, Málaga, Spain; 6 CIBER de Fisiopatología de la Obesidad y Nutrición (CIBEROBN), Universidad Jaime I, Castellón, Spain; 7 Servicio de Psiquiatría, Hospital Miguel Servet y Universidad de Zaragoza, Instituto Aragonés de Ciencias de la Salud, Zaragoza, Spain; University G. D’Annunzio, Italy

## Abstract

**Introduction:**

This work aimed to determine whether ^1^H magnetic resonance imaging (MRI), magnetic resonance spectroscopy (MRS), diffusion-weighted imaging (DWI) and diffusion tensor imaging (DTI) are correlated with years of meditation and psychological variables in long-term Zen meditators compared to healthy non-meditator controls.

**Materials and Methods:**

*Design.* Controlled, cross-sectional study. *Sample.* Meditators were recruited from a Zen Buddhist monastery. The control group was recruited from hospital staff. Meditators were administered questionnaires on anxiety, depression, cognitive impairment and mindfulness. ^1^H-MRS (1.5 T) of the brain was carried out by exploring four areas: both thalami, both hippocampi, the posterior superior parietal lobule (PSPL) and posterior cingulate gyrus. Predefined areas of the brain were measured for diffusivity (ADC) and fractional anisotropy (FA) by MR-DTI.

**Results:**

Myo-inositol (mI) was increased in the posterior cingulate gyrus and Glutamate (Glu), N-acetyl-aspartate (NAA) and N-acetyl-aspartate/Creatine (NAA/Cr) was reduced in the left thalamus in meditators. We found a significant positive correlation between mI in the posterior cingulate and years of meditation (r = 0.518; *p* = .019). We also found significant negative correlations between Glu (r = −0.452; *p* = .045), NAA (r = −0.617; *p* = .003) and NAA/Cr (r = −0.448; P = .047) in the left thalamus and years of meditation. Meditators showed a lower Apparent Diffusion Coefficient (ADC) in the left posterior parietal white matter than did controls, and the ADC was negatively correlated with years of meditation (r = −0.4850, *p* = .0066).

**Conclusions:**

The results are consistent with the view that mI, Glu and NAA are the most important altered metabolites. This study provides evidence of subtle abnormalities in neuronal function in regions of the white matter in meditators.

## Introduction

In current research contexts, mindfulness is defined as nonjudgmental attention to experiences in the present moment [1]. Mindfulness is cultivated in formal meditation practices, such as sitting meditation, walking meditation and mindful movements [2]. Mindfulness meditation has beneficial effects on a number of psychiatric, functional somatic, and stress-related symptoms and, therefore, has been increasingly incorporated into psychotherapeutic programs [2], [3].

However, how does meditation work? The scientific bases of mindfulness involves attention, body awareness, the regulation of emotion, changes in self perspective and the neural modulation of specific brain areas, including the anterior cingulate cortex (ACC), posterior cingulate cortex, (PCC), medial prefrontal cortex (MPFC), insula, temporo-parietal junction (TPJ), hippocampus, and amygdala [4].

Several neuroimaging studies support these data. Many papers are appearing that show differences in grey matter and/or white matter, and even longitudinally measured changes in meditators’ brains. For instance, in an analysis of brain grey matter, cortical thickness in the dorsal anterior cingulate cortex (ACC) was greater in experienced meditators compared with control subjects [5]. Cross-sectional studies that compared mindfulness meditators and non-meditators found that meditators showed greater grey matter concentration in the hippocampus [6], [7]. In an examination of participants who underwent mindfulness-based stress reduction, structural changes in the hippocampus were detectable within a period of only 8 weeks [4]. In addition, the cumulative hours of meditation training have been positively correlated with grey matter concentration in the ventromedial PFC in experienced meditators [8]. Functional MRI studies found that individuals who had completed a mindfulness-based stress reduction course demonstrated increased insula activation when they focused on their momentary experience compared with individuals who had not practiced mindfulness [9]. This study also found increased activation of the secondary somatosensory area, which is relevant for the processing of exteroceptive sensory events [9]. In addition, during mindfulness meditation, experienced mindfulness meditators show greater activation in the dorso-medial PFC and rostral ACC compared with non-meditators [9]. After participants completed an 8-week mindfulness-based stress reduction course, Farb et al. [10] found increased activity in participants’ ventrolateral PFC, which the authors interpreted as augmented inhibitory control. Recently, a positive association between the describing facet of mindfulness and gray matter volume in the right anterior insula and the right amygdala has been described [11].

Functional MRI (fMRI) is a neuroimaging technique employed to investigate cerebral connectivity. Most fMRI studies have analyzed brain activity upon the execution of multimodal tasks. More recently, fMRI has also been used to evaluate neural activity in resting conditions and the related multiple functional networks (i.e.: sensory-motor, visual, auditory, attention, language, and default networks) [12].

The default mode network (DMN) comprises a set of brain regions that are co-activated during passive task states, show an intrinsic functional relationship, and are connected via direct and indirect anatomic projections. The medial temporal lobe subsystem provides information from previous experiences in the form of memories and associations, which are the building blocks of mental simulation. The medial prefrontal subsystem facilitates the flexible use of this information during the construction of self-relevant mental simulations. These 2 subsystems converge on important nodes of integration, including the posterior cingulate cortex (PCC) [13]. The medial temporal lobe subsystem includes both the hippocampal formation (HF) and parahippocampal cortex (PHC). This subsystem has been associated with key hubs of the default network, including the posterior cingulate/retrosplenial cortex (PCC/Rsp), ventral medial prefrontal cortex (vMPFC), and inferior parietal lobule (IPL).

Magnetic resonance spectroscopy is a non-invasive and analytic method for the detection of metabolites present in an operator selected area of the brain (ROI: region of interest). The most commonly evaluated metabolites are N-acetyl-aspartate (NAA), myo-inositol (mI), choline (Ch), creatine (Cr) and glutamate (Glu). Creatine is used as internal reference value, since it is the most stable cerebral metabolite. Ratios between metabolites and creatine are also of great value as they counteract the systematic errors of measurements. N-acetylaspartate is one of the most abundant amino acids in the central nervous system, located predominantly in neurons, axons and dendrites. The sugar-alcohol compound myo-inositol may act as an osmoregulator, intracellular messenger and detoxication agent; it is also regarded as a marker of glial cells [14].

Glx (Glu+Gln) is an excitatory aminoacid, and excessive Glx neurotransmission has been implicated in excitotoxic neuronal damage [15]. To our knowledge, there are no other MRS studies of meditation.Water molecules in the brain are in constant Brownian motion, and although the movement of theses protons affects conventional structural imaging, diffusion weighted imaging (DWI) and diffusion tensor imaging (DTI) allow quantification of this microscopic movement within each voxel. The appropriate mathematical combination of the directional diffusion-weighted images provides quantitative measures of water diffusion for each voxel via the apparent diffusion coefficient (ADC), as well as the degree of diffusion directionality, or fractional anisotropy (FA) [16]. Grey matter is composed of neuronal cell bodies and dendrites concentrated in the outer layers of the cortex. Microstructural changes in white matter can be revealed by specialized MRI brain imaging techniques such as diffusion tensor imaging (DTI). This method analyses proton diffusion in tissue, which is more restricted in white matter than in grey matter. FA augments with increased myelination, diameter and axon compaction. Although the adult brain was once seen as a rather static organ, it is now clear that the organization of brain circuitry is constantly changing as a function of experience or learning [17].

Luders et al. showed with diffusion tensor imaging (DTI), pronounced structural connectivity throughout the entire brain within major projection pathways, commissural pathways, and association pathways in meditators compared to controls [18]. Recently, Tang found that a form of mindfulness meditation, integrative body-mind training (IBMT), improved FA in areas surrounding the anterior cingulate cortex after 4-week training more than in controls given relaxation training. Reductions in radial diffusivity (RD) have been interpreted as improved myelin but reductions in axial diffusivity (AD) involve other mechanisms, such as axonal density [19].

In the present study, we performed magnetic resonance spectroscopy (MRS), diffusion weighted imaging (DWI) and diffusion tensor imaging (DTI) in meditators and in non-meditator healthy controls to evaluate whether these neuroradiological techniques discriminate the brain patterns of the two groups and to elucidate the possible association between meditators’ brain changes and years of meditation.

## Methods

### Design

Case-control study.

### Patients

The group of meditators (N = 10) was recruited from the Soto Zen Spanish Buddhist community. Individuals were required to meet the following inclusion criteria: 18 to 65 years old; able to understand Spanish; long-term meditative practice (>8 years meditating for an average of 1 hour daily); no psychiatric disorder or pharmacologic treatment one year before the study began. The healthy control group (N = 10) was recruited among hospital staff (comprising 4,800 workers: 700 doctors, 2,600 non-medical health professionals and 1,500 administrative and services personal), with an adjustment for gender, age (+/−3 years), years of education (+/−3 years) and ethnic group.

The study was approved by the Aragon Ethics Committee and performed in accordance with the ethical standards of the 1964 Declaration of Helsinki. All participants gave written informed consent prior to their inclusion in the study.

### Measurements

#### Sociodemographic and clinical variables

Sociodemographic data: Gender, age, marital status, education, and occupation were collected.

Hospital Anxiety Depression Scale (HADS) [20]: This is a self-report scale designed to screen for the presence of depression and anxiety disorders in medically ill patients. HADS was used for the analysis of this sample of healthy people, as this questionnaire has been recommended for use in community studies and primary care settings. It contains 14 items that are rated on a 4-point Likert-type scale. Two subscales assess depression and anxiety independently (HADS-Dep and HADS-Anx, respectively) [20]. The Spanish validated version was used [21].

Mini-Mental State Examination (MMSE) [22]: This is a fully structured scale that includes the following seven categories: orientation to place, orientation to time, registration, attention and concentration, recall, language, and visual construction [23].

Mindful Attention Awareness Scale (MAAS) [24]: The MAAS is a 15-item scale designed to assess a core characteristic of dispositional mindfulness, namely, open or receptive awareness of and attention to what is taking place in the present. The scale shows strong psychometric properties and has been validated with college, community, and cancer patient samples. There is a Spanish version of the MAAS that displays adequate psychometric properties [25].

#### Neuroimaging techniques

**Figure 1:**
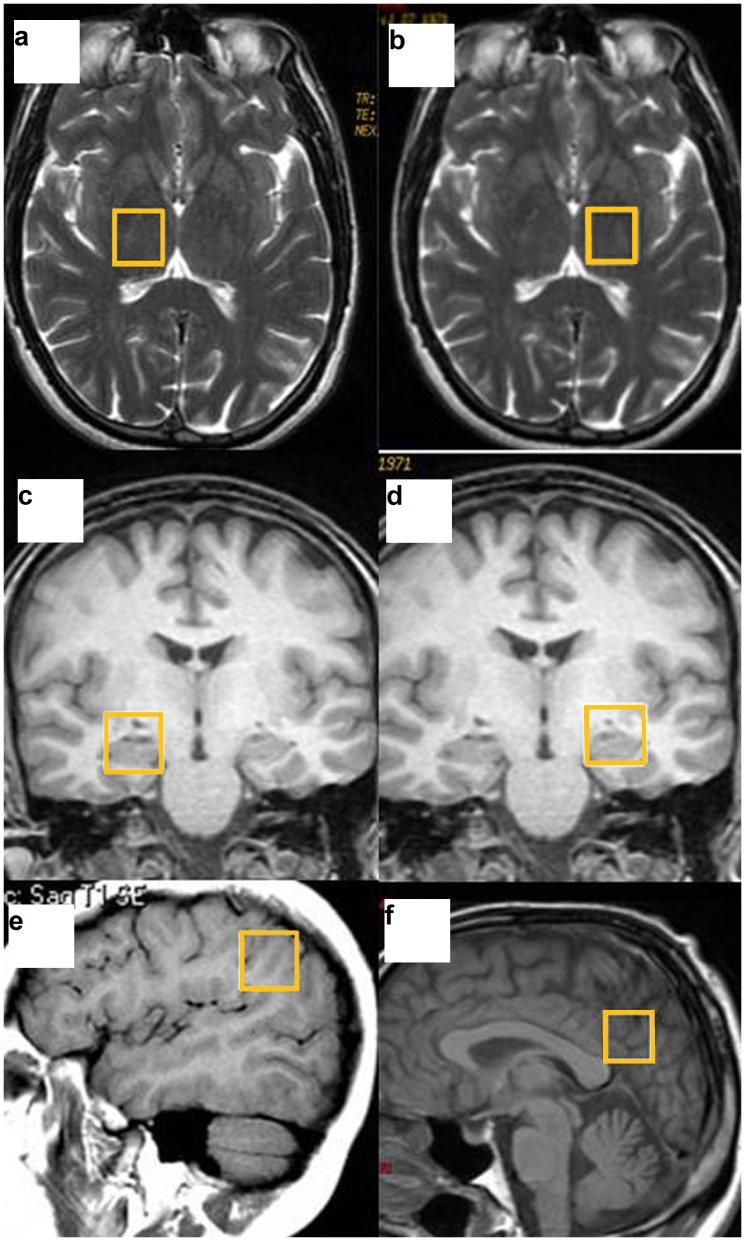
Voxel placement in the different brain regions. The thalamus (**a,b**), hippocampus (**c,d**), posterior superior parietal lobule (**e**) and posterior cingulate gyrus (**f**).

All patients underwent the following neuroimaging techniques:

1: Magnetic resonance imaging (MRI): Data were acquired using a 1.5-T Signa HD clinical scanner (GE Healthcare Diagnostic Imaging, Milwaukee, WI, USA). All images were acquired using an eight-channel phased array head coil (NVHEAD A).
2: Magnetic resonance spectroscopy (MRS): The basic principle underlying single-voxel localization techniques is to use three mutually orthogonal slice selectivepulses and design the pulse sequence to collect only the echo signal from the point (voxel) in space where all three slices intersect. An axial T2-weighted image was used to locate volumes of interest (VOIs) (2×2×2 cm) in both halves of the thalamus. A coronal T2-weighted image in the plane that goes through the inner auditory conducts and brain peduncle was used to locate volumes in both hippocampi, and a sagittal T1-weighted image was used to locate a voxel on the posterior superior parietal lobule (PSPL) and posterior cingulate gyrus (Figure 1). For the quantitative regional analysis, based on the Talairach Atlas [26], the ROI acquisition was based on the plane formed by the anterior and posterior commissures (APCP). The thalamus was measured at 5 mm above the APCP. A sagittal T1-weighted image was used to locate a voxel on the posterior superior parietal lobule (PSPL) and posterior cingulate gyrus (drawing a line perpendicular to the splenium of the corpus callosum and another line oblique to the surface of the corpus callosum. The intersection of the two lines was positioned above the lower corner of the voxel). The plane to measure both hippocampi was 20 mm forward of the coronal plane passing through the splenium of the corpus callosum. 1H-MRS was carried out using a short echo time (TE) of 35 msec and a repetition time (TR) of 2,000 msec and 128 accumulations using a single-voxel stimulated-echo acquisition-mode localization sequence with a spin-echo technique that uses selective excitation with gradient spoiling for water suppression. The mode of spectral acquisition was probe-p (PRESS technique). Chemical concentrations can now be automatically extracted from MR spectra using sophisticated and well-documented time-domain and spectral frequency fitting software packages such as LCModel (Stephen Provencher, Oakville, Ontario, Canada), a user-independent fitting routine based on a library of model spectra of all individual metabolites.

Concentration values are expressed as arbitrary institutional units and are not corrected for contributions by cerebrospinal fluid (CSF) or a small reduction in the numeric values due to residual T1 and T2 relaxation effects. The data evaluation comprised a correction of the spectroscopic time-domain data for residual eddy-current effects.

**Figure 2:**
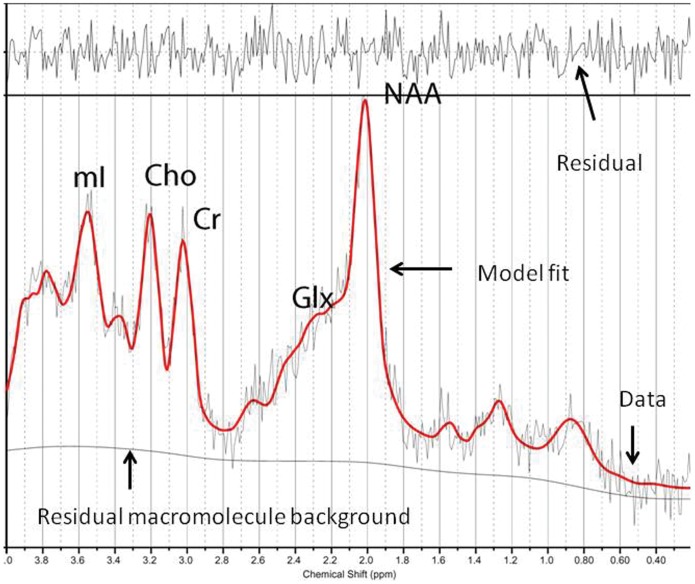
Representative left thalamus spectrum. The narrow line width indicates excellent data quality and the uniform residual represents excellent model fit. NAA: N-acetyl-aspartate; Glx: glutamate+glutamine+GABA; Cho: Choline; Cr: creatine+phosphocholine; mI: myo-inositol.

Quantifiable chemicals by MRS included the following: *N*-acetylaspartate (NAA), 2.02 ppm; glutamine (Gln), glutamate (Glu) and Gln+Glu (Glx), 2.1 to 2.55 ppm; total creatine (Cr; composed of creatine and phosphocreatine), 3.03 ppm; choline-containing compounds (Cho), 3.23 ppm; and myo-inositol (mI), 3.56 ppm (Figure 2). We also obtained the ratios of the peak amplitude of the metabolites relative to creatine. The areas of exploration were chosen based on brain structures that are activated during meditation conditions (thalamus) [27], that demonstrate decreased activity during intense meditation (posterior superior parietal lobule) [28], [29], and that modulate and moderate cortical arousal and interconnections with other neocortical areas (hippocampus, posterior cingulate gyrus) [30]. Prior to the current study, we examined the test-retest reliability of metabolite measurements in every area in a sample of patients through two consecutive studies, without removing the patient from the scanner. According to the resultant α coefficients, we must assume a mean random variation in the posterior gyrus of around 8% for mI/Cr and of around 10% for NAA/Cr, Cho/Cr, and Glutamate [31].

3: Diffusion weighted imaging (DWI) and Diffusion tensor imaging (DTI): 650 images were obtained using a single-shot, spin-echo EPI technique with a b-value of 1,000 s/mm2 for each of 25 diffusion encoding directions, TE = 94.5 ms, TR = 8,000 ms, matrix = 128×128, field of view, 24 cm, 3-mm slice thickness with no gaps, 25 slices, number of excitations 2 and a scan time of 7.25 min. The diffusion MR data were analysed using the diffusion tensor model. After a mathematical diagonalization process, the eigenvectors and eigenvalues that describe the tensor ellipsoid were determined. Subsequently, two standard diffusion indices were derived, mean diffusivity (MD) or the Apparent Diffusion Coefficient (ADC) and the fractional anisotropy (FA) [13], [32]. The ADC and FA maps were calculated off-line with the Functool software 3.1.23 in the Advantage Workstation 4.3 (General Electric Medical Systems, Milwaukee, WI) in accordance with the following procedure. Initially, images were preprocessed to remove image-to-image misregistration that arises from directional eddy currents during echo-planar readout. Directional diffusion weighted images (DWIs) were spatially registered to the b ≈ 0 image, which was set to remove image shear, compression, and shift by an affine transform. ADC is considered quantitative with normal brain values of ADC = 0.7×10−3 mm^2^/sec, and FA is a dimensionless value between zero (isotropic) and close to 1 (highly anisotropic environments).

**Figure 3:**
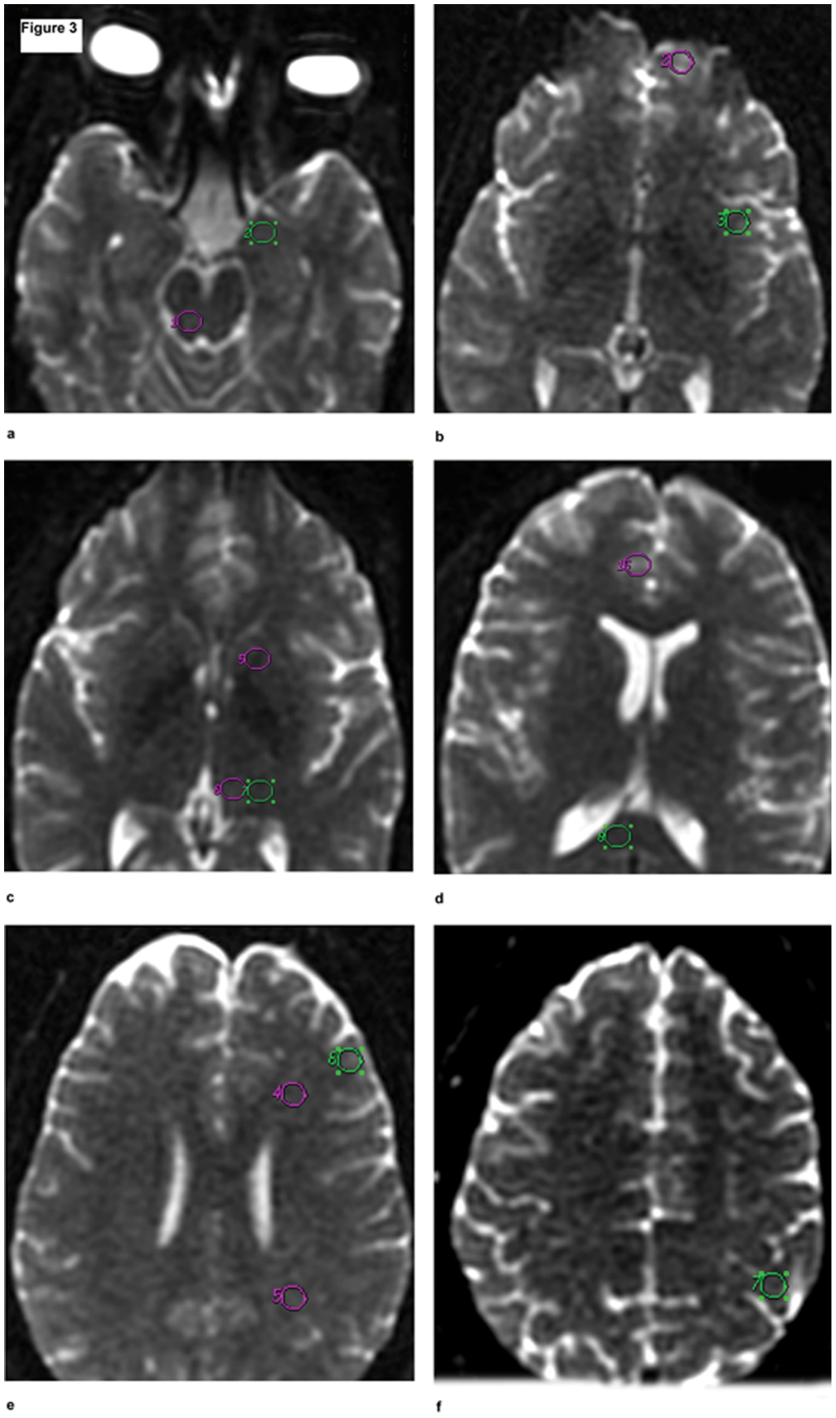
Axial diffusion images showing the different locations of the equal-sized regions of interest (ROIs) were placed in normal appearing brain parenchyma. The ROI placements for periaqueductal grey (1) and amygdale (2) (**a**); orbital cortex (2) and insular cortex (3) (**b**); internal capsule (5), thalamus ventral (6) and dorsal (7), (**c**); cingulate gyrus cortex (16) and corpus callosum (8), (**d**); frontal white matter (4), parietal white matter (5), and dorsolateral prefrontal cortex (6) (**e**); and left motor cortex (7) (**f**).

Standardised 50-mm^3^ circular regions of interest (ROIs) were placed at the following areas: periaqueductal grey and amygdale, orbital cortex, insular cortex, internal capsule, thalamus ventral and dorsal, cingulate gyrus cortex, corpus callosum, frontal white matter, parietal white matter, dorsolateral prefrontal cortex and left sensorimotor cortex (Figure 3). The mean ADC and FA in the different regions were compared between the two groups. The total scanning time for six MRS acquisitions (30∶20), T1- (1∶39) and T2-weighted scans (1∶11), and DWI (7.25) was 40∶34.

### Statistical analysis

To describe the quantitative variables, the means and standard deviations were calculated. The sociodemographic variables were compared by group using the chi-squared or U Mann-Whitney test, depending on whether the variable was categorical or numerical, respectively. To analyse possible differences in brain metabolite levels between meditators and healthy non-meditator controls, paired t-tests were utilised (due to matching between both groups in relevant variables such as age, gender, ethnic group and years of education). In addition, we used nonparametric Spearman's rho correlation (due to samples not fulfilling the assumption of normality) to study the relationship between brain metabolites for which the levels were significantly different and years of meditation and psychological variables. Statistical analyses were conducted using SPSS 15.0, and *p* values lower than 0.05 were considered statistically significant for all analyses. Owing to multiple comparisons in brain metabolite levels between meditators and healthy non-meditator controls, statistical significance was placed at 0.01.

## Results

### Sociodemographic and Psychological Variables

Ten meditators (8 men and 2 women) were compared to 10 matched healthy non-meditator control subjects. No individuals in either of the two groups were excluded during recruitment. No significant differences were found in age (meditators: 39.52, SD: 11.13; non-meditators: 44.51, SD: 9.58; *p* = .318), years of education (meditators: 12.0; SD: 2.44; non-meditators: 12.10; SD: 2.28; *p* = .876) or ethnic group (all meditators and controls were European) among the groups, as was expected due to matching.

In both groups, rating scores on the psychopathology questionnaires (anxiety, depression and cognitive function) were within the normal ranges; however, ratings of anxiety and depression (measured with HADS) were significantly lower in the meditator group (HADS-anx: 0.30, SD = 0.48 in meditators vs 1.70; SD = 0.94 in non-meditators, *p*<0.05; HADS-dep: 0.30; SD = 0.48 in meditators vs 1.60; SD = 0.51 in non-meditators, *p*<0.05). There was no difference in cognitive function. Mindfulness, as measured by MAAS, was significantly higher in meditators, as was expected due to years of meditation in this group (range: 96–360 months) (Table 1).

**Table 1:** Comparison of psychological variables in non-meditator healthy controls and meditators.

Psychological variables	Non-meditator healthy controls (N = 10)	Meditators (N = 10)	Significance
Anxiety (HADS-anx) (mean, SD)	1.70 (0.94)	0.30 (0.48)	*p* = 0.002
Depression (HADS-dep) (mean, SD)	1.60 (0.51)	0.30 (0.48)	*p* = 0.001
Cognitive function (MMSE) (mean, SD)	35 (0)	35 (0)	*p* = 1
Mindfulness (MAAS) (mean, SD)	19.1 (2.96)	74.1 (5.32)	*p* = 0.001
Months of meditation in meditators (mean, SD, range)	––––––	190.80 (91.81); range: 96–360 months	*–––*

Mann-Whitney U.

### Neuroimaging Variables

#### MRI

The conventional MR images showed the absence of T2 hyperintensities and morphological alterations in all subjects.

#### MRS

**Figure 4:**
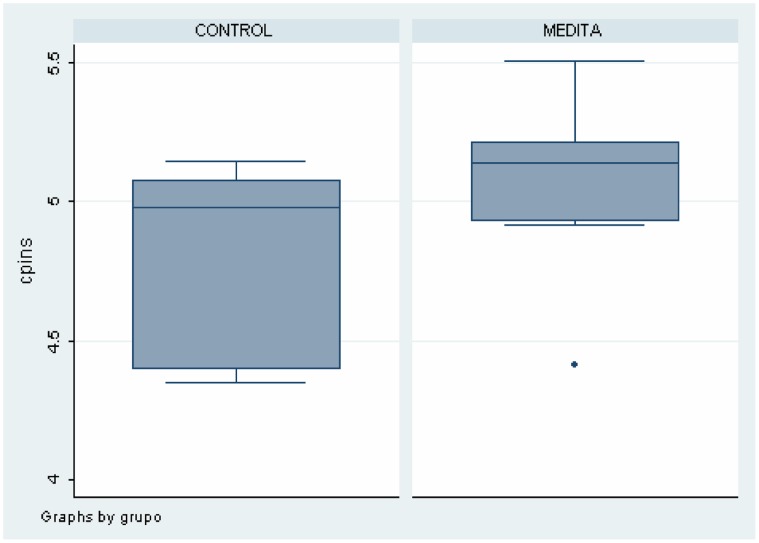
Differences on Myo-Inositol levels in Posterior Cingulate between meditators and healthy non-meditators.

**Figure 5:**
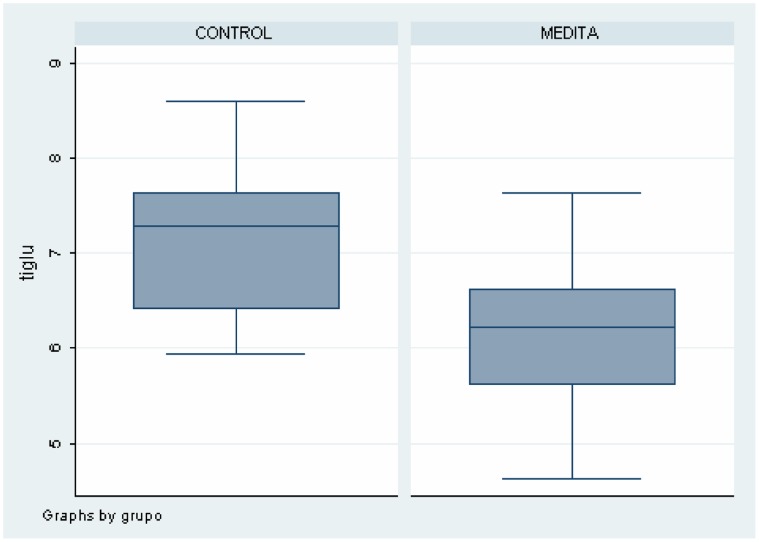
Differences on Glutamate levels in Left Thalamus between meditators and healthy non-meditators.

**Figure 6:**
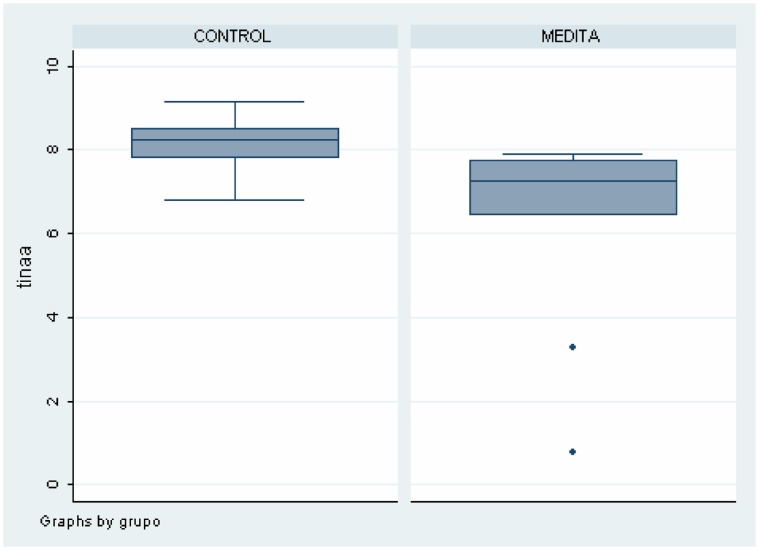
Differences on NAA levels in Left Thalamus between meditators and healthy non-meditators.

**Figure 7:**
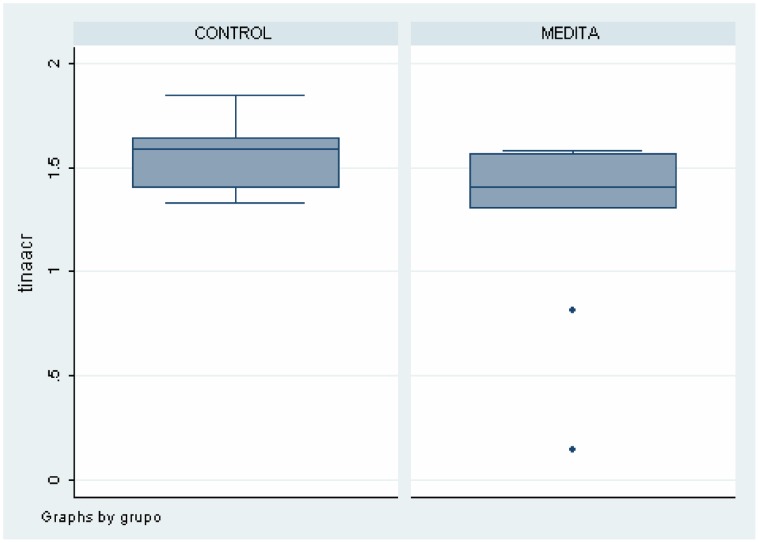
Differences on NAA/Creatine in in Left Thalamus between meditators and healthy non-meditators.

As shown in Table 2, there were differences between meditators and healthy non-meditators in several brain areas and metabolites. These data can also be seen in Figures 4, 5, 6, 7. Meditators showed increased myo-inositol in the posterior cingulate gyrus (*p* = .003) and decreased Glutamate (*p* = .004), N-acetyl-aspartate (*p* = .003) and N-acetyl-aspartate/Creatine (*p* = .002) levels in the left thalamus. We found a significant positive correlation between mI in the posterior cingulate cortex and years of meditation (r = 0.518; *p* = .019) (Table 3). We also found significant negative correlations between Glu (r = −0.452; *p* = .045), NAA (r = −0.617; *p* = .003) and NAA/Cr (r = −0.448; *p* = .047) in the left thalamus and years of meditation (Table 3). No correlation was found with respect to the other metabolites in any of the areas. Differences in metabolite levels between meditators and healthy non-meditators remained after adjusting by depression and anxiety using the analysis of covariance (ANCOVA) test.

**Table 2:** Comparison of Metabolite Levels and Metabolite/Creatine ratios in Posterior Cingulate and Left Thalamus determined by Proton Magnetic Resonance Spectroscopy in non-meditator healthy controls and meditators.

Region and Metabolites	Non-meditator healthy controls (N = 10)	Meditators (N = 10)	
	Mean SD	Mean SD	*p*
***Posterior Cingulate***			
Myo-Inositol	4.82 (0.32)	5.08 (0.29)	0.003
***Left Thalamus***			
Glutamate	7.17 (0.80)	6.23 (0.93)	0.004
NAA	8.19 (0.70)	6.30 (2.37)	0.003
NAA/Cr	1.56 (0.17)	1.26 (0.45)	0.002

**Table 3:** Correlations among brain metabolites in MRI and years of meditation.

Region and Metabolites	Correlation (r)	*p*
***Posterior Cingulate***		
Myo-Inosotol	0.518	0.019
***Left Thalamus***		
Glutamate	−0.452	0.045
NAA	−0.617	0.003
NAA/Creatine	−0.448	0.047

#### DWI and DTI

*a)* Apparent Diffusion Coefficient (ADC). Meditators showed a lower mean diffusivity (MD i.e., ADC) in the left posterior parietal white matter (6.59e^−10^; SD: 1.49e^−11^) than did controls (7.03e^−10^; SD: 1.17e^−11^), and this diffusivity was correlated with years of meditation (r = −0.485, *p* = .0066) (Table 4). There were no other significant differences in the ADC between meditators and non-meditator controls. b) Fractional anisotropy (FA). In comparison with healthy non-meditator controls (0.219; SD: 0.017), meditators evidenced decreased FA values (0.170; SD: 0.016) in the grey matter of the left primary sensorimotor cortex. These FA values did not correlate with years of meditation. Moreover, there were no significant differences in FA measurements between the right and left brain hemispheres in the meditators or in non-meditator controls.

**Table 4:** Comparison of findings in Diffusion Tensor Imaging in non-meditator healthy controls and meditators.

Technique and brain region	Non-meditator healthy controls (N = 10)	Meditators (N = 10)	
	Mean SD	Mean SD	*p*
***Apparent Diffusion Coefficient***			
Left posterior parietal white matter	7.03e^−10^ (1.17e^−11^)	6.59e^−10^ (1.49e^−11^)	0.0257
	(Correlation with length of meditation: r = −0.485; *p* = 0.006)		
***Fractional Anisotropy***			
Left primary sensorimotor cortex	0.219 (0.017)	0.170 (0.016)	0.0451
	No correlation with years of meditation		

Mann-Whitney U.

## Discussion

The present study is, to our knowledge, the first study to utilise magnetic resonance spectroscopy to evaluate the brain metabolite patterns in long-term meditators, to compare them with healthy non-meditating individuals and to elucidate the possible association between meditators’ brain changes and years of meditation. In the present study, we found that meditation was associated with significantly higher mI in the posterior cingulate gyrus and decreased Glu, NAA and NAA/Cr in the left thalamus. In addition, meditators showed a lower apparent diffusion coefficient in the left posterior parietal white matter than did controls. These findings occurred despite comparable age, education and neuropsychological test performance between the two groups, and all of these changes in meditators were correlated with years of meditation. These results are consistent with the idea that meditation can initiate changes in cerebral metabolism as well as other axonal changes in adults who meditate. In the next section, we will discuss the implications of these findings.

### Findings on MRS

#### Increased mI in the posterior cingulate gyrus

Inositol is a simple isomer of glucose that is a key metabolic precursor in the phosphatidylinositol cycle. Unlike L-dopa and tryptophan, which are amino acid precursors of monoamine neurotransmitters and which have been reported to have antidepressant properties, inositol is a precursor of an intracellular second messenger system [33]. Barkai et al. [34] reported that depressed patients, both unipolar and bipolar, had markedly low levels of inositol in CSF. Levine et al. [35] showed that inositol treatment led to a decline in mean Hamilton Depression Rating Scale score and had a significant antidepressant effect [36].

Elevated concentrations of cerebral mI have been extensively reported in conditions associated with cognitive impairment such as Down’s syndrome [37], Alzheimer’s disease [38] and amnestic Mild Cognitive Impairment [39]. Increased cerebral mI levels have also been reported in patients with diabetes mellitus [40] and fibromyalgia [41]. Therefore, the current finding of increased mI in the posterior cingulate gyrus in long-term meditators seems counterintuitive.

Changes in mI concentrations might reflect disturbances in fluid homeostasis and cellular signalling. It has been proposed as a glial marker [42] and is thought to reflect the degree of glial activation [43] and to be stored in microglia before being used in the PI-cycle in neurons [44].

Microglia has the ability to express the fully functional interleukin IL-2 receptor [45]. Interleukin (IL)-2 regulates the immune response through the proliferation of activated T-cells and affects the central nervous system (CNS). In addition to marked neurobehavioral effects, IL-2 impacts various psychiatric disorders. The immune-CNS communication of IL-2 remains unclear, although, it has been suggested that microglia are the source and target of IL-2. Peripherally administered IL-2 impacts CNS metabolites. In vivo 1H magnetic resonance spectroscopy (MRS) revealed a significant increase of myo-inositol in the PFC and hippocampus. This evidence strongly suggests that the metabolite changes observed here are likely due to the effects of IL-2.

The precise mechanism of action on the CNS is not yet known, but the evidence presented here implicates the activation of microglia following the peripheral injection of IL-2. In addition, there is evidence that IL-2-induced neurochemical changes might have a delayed functional relevance for affective conditions, such as anxiety-like behaviour. Consistent with this assumption, cytokines modulate serotonergic neurotransmission and enhance the catabolism of tryptophan (the base amino acid of serotonin), leading to a reduction in the levels of serotonin and an increase in tryptophan catabolites.

Schneider et al. [46] examined the reactivity of microglia in response to interleukin IL-2, and their results support the view that microglia act as part of a mechanism by which peripheral immune signals could affect centrally mediated affective behaviours. A significant correlation between anxiety-like measures and myo-inositol, a marker for microglia activity, was found in the hippocampus. The fluorescence activated cell sorting (FACS) analysis showed a significant increase in CD25 (+) microglia in the hippocampus compared to controls. The results support the role of microglia as a mediator in the relationship between immune-CNS communication and peripheral IL [47].

#### Low glutamate and NAA in the left thalamus

The lateral posterior nucleus of the thalamus provides the posterior superior parietal lobule (PSPL) with the sensory information it requires to determine the body’s spatial orientation. Due to increased activity in the prefrontal cortex (PFC) during meditation, a concomitant increase in the activity in the reticular nucleus of the thalamus should be evident [27].

The PSPL is heavily involved in the analysis and integration of higher-order visual, auditory, and somaesthetic information [48]. It is also involved in a complex attentional network that includes the PFC and thalamus [49]. Deafferentation of the PSPL has also been supported by two imaging studies that demonstrated decreased activity in this region during intense meditation [28], [29].

Furthermore, one SPECT study showed a significant correlation between increased activity in the thalamus and decreased activity in the PSPL. The hippocampus acts to modulate and moderate cortical arousal and responsiveness via rich and extensive interconnections with the prefrontal cortex, other neocortical areas, the amygdala, and the hypothalamus [30]. The ability of the hippocampus to stimulate or inhibit neuronal activity in other structures likely relies upon the glutamate and GABA systems, respectively [50].

The dopaminergic system, via the basal ganglia, is believed to participate in the regulation of the glutamatergic system and the interactions between the prefrontal cortex and subcortical structures. A recent PET study during the practise of Yoga Nidra meditation demonstrated a significant increase in dopamine levels. The experimenters hypothesised that this increase may be associated with the gating of cortical–subcortical interactions, leading to the overall decrease in readiness for action that is associated with this particular type of meditation [51].

Glutamate is the primary excitatory neurotransmitter in the brain; therefore, glutamate-mediated synaptic transmission is critical for brain functions [52]. Thus, it has been proposed that the physiological responses seen on functional MRI and PET [53] that arise from increased energy demand with neuronal activation are directly related to this glutamate/glutamine (Glx) cycle.

The N-methyl-D aspartate (NMDA) receptor, which has a high affinity for glutamate, is also widely distributed throughout the brain. It has been shown that the glutamatergic system is involved in excitatory synaptic transmission, plasticity, and excitotoxicity in the central nervous system [54]. Moreover, signal transmission malfunction at the NMDA receptor site has been implicated in several neurological and psychiatric disorders, such as Alzheimer’s disease and schizophrenia, [55] which may be due to altered glutamate concentration.

Rothman et al. [56] proposed that the physiological responses observed in fMRI that arise from increased energy demand with neuronal activation are directly related to the Glx cycle. A glutamatergic effect in the forced-attention paradigm has not previously been demonstrated and could have implications for understanding the neurophysiological underpinnings of higher cognitive functions, such as cognitive control, and for PFC functioning [60]. Fonnum et al. [57] showed that corticostriatal and cortico-thalamic fibres have an active high affinity uptake of D-Asp and contain a high level of endogenous glutamate. They suggested that acidic amino acids, particularly glutamate, should be regarded as strong transmitter candidates for these corticofugal systems.

Glutamate activates N-methyl-D-aspartate receptors (NMDAr), but excess glutamate can kill these neurons through excitotoxic processes. If glutamate levels approach excitotoxic concentrations during intense states of meditation, the brain may limit its production of N-acetylated-a-linked-acidic dipeptidase, the enzyme responsible for converting the endogenous NMDAr antagonist N-acetylaspartylglutamate (NAAG) into glutamate [58]. The glutamate in frontal circuits is an important regulator of dopamine [59]. Via a feedback mechanism, dopamine concentration may influence the concentration of glutamate [60], [61]. MRS-detectable decreases in Glu content may be a consequence of a change in metabolic activity reflecting decreased function or viability of neurons because Glu, similar to NAA, is located primarily in neurons [62].An important consideration in the interpretation of these results is that the MRS-detectable Glu signal does not discriminate between the metabolic and neurotransmitter pool of Glu. It has been estimated that 70–80% of tissue Glu is present in the metabolic pool and 20–30% in glutamatergic nerve terminals, and it is not known whether the elevation of Glu occurs in the neurotransmitter pool. Moreover, the cellular compartment originating the elevated Glu signal remains unknown.

Spectroscopy revealed a reduction in the concentration of N-acetyl-aspartate (NAA) and N-acetyl-aspartate/Creatine (NAA/Cr).This reduction might reflect a combination of the loss of neural cells, reduced neural metabolism, loss of dendritic structures, and reduced myelination. Reduced NAA or NAA/Cr levels have typically been attributed to a reduction in the neuronal density or neuronal function within the VOIs [63], as NAA is almost entirely located within neurons in the CNS. The reduced neuronal density might reflect neuronal death or decreased tissue volume. The reduced NAA signal could be interpreted as a sign of neuronal dysfunction and does not necessarily indicate cell death. This interpretation is consistent with that of Stanley et al. [64], who observed a reduction of free-PME in the prefrontal regions and basal ganglia.

One study showed decreased NAA levels in the left temporal lobe and left cerebellum in persons with autism [65]. Another found low cerebellar NAA levels in persons with autism and other disorders [66]. Within the mitochondria of neuronal bodies, aspartic acid is acetylated. The function of NAA within axons in the white matter is unknown, but one of its roles may involve the synthesis of neurotransmitters [67]. One question remains as to whether the depletion of NAA levels could signify a decreased activation of inhibitory neuronal pathways in meditators. The depletion of NAA concentration could reflect decreased mitochondrial metabolism, which might correlate with years of meditation.

#### Findings on DWI and DTI

Microstructural changes in white matter can be revealed by specialised MRI brain imaging techniques such as diffusion weighted imaging (DWI) and diffusion tensor imaging (DTI). These methods analyse the of proton diffusion in tissue, which is more restricted in white matter than in grey matter. The anisotropy increases with increased myelination, diameter and axon compaction.

#### Lower mean diffusivity in the left parietal white matter

In the current study, meditators showed a significantly lower mean diffusivity (MD) (i.e., ADC) in the left parietal white matter (WM) than did control, and the MD was correlated with time of meditation. Diffusion weighted imaging (DWI) is a structural method that assesses the microscopic translational movement of molecules via thermally driven random, so-called Brownian, motion of water molecules. To quantify the degree of water movement, it is necessary to process images and obtains diffusion maps called “Apparent Diffusion Coefficient (ADC)”. Unlike DWI, the areas with restricted movement of water have low values of ADC in these maps. ADC/MD values are reduced during brain myelination. The reduced diffusion in the white matter during brain maturation has been initially explained in terms of the development of myelin, which acts as a barrier to diffusion. Diffusion-weighted MR imaging in the maturing brain has confirmed this diffusion restriction [68]. Thus, the reduction in the ADC values during brain myelination must be further explained. In addition, the water loss in the developing brain [69], early wrapping of axons during the oligodendroglial process [70], increasing macromolecule concentration, increased membrane surface-to-cell volume ratio [71], increased axonal diameters and increase in microtubule-associated proteins [72] must be addressed. In adults older than 40 years, high ADC values were observed in the cerebral white matter with increasing age.

Using diffusion tensor imaging, several recent studies have shown that training induces changes in white matter efficiency, as measured through fractional anisotropy (FA) [73]. In the present study, we observed that the ADC values are a crude measure of the barrier density and cellularity of denser tissues [74].

Our results demonstrate the time-course of white matter neuroplasticity in long-term meditation. The increased myelination would enhance communication among cortical areas, resulting in enhanced performance. A previous study showed that 4 weeks of integrative body–mind training (IBMT) (11 h in total) enhanced FA in several brain areas involved in the communication to and from the anterior cingulate cortex (ACC), including the corpus callosum and anterior and superior corona radiate [75].

Our study also showed a negative correlation between the lower Apparent Diffusion Coefficient (ADC) in the left posterior parietal white matter and years of meditation. Thus, the improved self-regulation following IBMT might be mediated through the increased communication efficiency between the left posterior parietal and other brain areas. Thus, these results imply that the enhanced integrity of white matter fibres through long-term meditation might reflect the increased numbers of brain fibres or increased axonal calibre. Increased myelination could occur as a consequence of increased neural firing in active brain areas during training [76].

Several studies have demonstrated that emotions and stress change white matter integrity [77], [78], possibly reflecting less automatic, mindless processing and more top-down control.

Recent evidence from anatomical MRI studies demonstrated that the aforementioned brain regions show structural changes following mindfulness meditation training. In cross-sectional studies comparing mindfulness meditators and non-meditators, meditators showed greater grey matter concentration in the hippocampus [6], [79]. Furthermore, Hölzel et al. [80] recently observed that structural changes in the hippocampus were detectable within a period of only 8 weeks in participants that underwent mindfulness-based stress reduction. Furthermore, Hölzel et al. [6] found that cumulative hours of meditation training were positively correlated with grey matter concentration in the vmPFC in experienced meditators.

Increased ADC (MD) has been described in multiple regions of white matter, corpus callosum, cingulum and hippocampus of patients with Alzheimer Disease as compared with controls [81] and patients with Lewy body dementia [63]. The increased diffusion in brains with AD has been attributed to the decrease of neurons, axons and dendrites, which leads to the expansion of extracellular space and quick diffusion of water [82].

In the present study, the primary somatosensory cortex as part of the postcentral gyrus, which receives the bulk of thalamo-cortical projections from the sensory input fields, showed no significant decrease in fractional anisotropy (FA) in meditators compared with age-matched non-meditators. The results only confirmed a non-significant trend of reduced anisotropy in the postcentral gyrus. Asymmetry of anisotropy has been reported in the superior longitudinal fasciculus [83], showing left greater than right asymmetry. Another study found that mindfulness meditators more robustly activated the left anterior, posterior, and mid-insula and the thalamus [84]. Recently, Luders et al. showed pronounced structural connectivity throughout the entire brain within major projection pathways, commissural pathways, and association pathways in meditators compared to controls. The largest group differences were observed within the corticospinal tract, the temporal component of the superior longitudinal fasciculus, and the uncinate fasciculus [16].

There is emerging evidence of cytokine–microglia interactions [85] and cytokine producing microglia that act upon neurons [86]. Thus, it is likely that neuro-immune contributions involving cytokine production/release by microglia impact neural networks and psychological disorders. The increased myo-inositol in default mode regions might indicate that the posterior cingulate cortex plays an important role to manipulate the functioning of the default mode network. We propose that the abnormalities observed in our study reflected the metabolism sensitivity of default mode regions.

The thalamus makes reciprocal connections with a wide area of the cortex. The reduced regional grey matter NAA might indicate handicapped neuron growth, neuronal death or an decreased tissue volume. Considering the general reduction in the NAA and NAA/Cr values, it is tempting to conclude that the functional magnetic resonance imaging studies of meditators have detected reduced neural activity due to a reduction in Glu, leading to neuronal hypoexcitability.

Our study also provides insight into brain plasticity, considering the age of our subjects and the remarkable changes in their brain during long-term meditation. These findings might serve as a vehicle for examining the behavioural consequences of different indices of white matter integrity, such as functional connectivity.

The newly identified modifications in the brain regions highlight the potential novel features and functions of these regions. Thus, our results demonstrate the mechanism of white matter neuroplasticity during long-term meditation.

Thus, MRS and DTI are excellent tools for examining training-related plasticity and the neural mechanisms underlying meditation.In summary, we demonstrated that long-term meditation is accompanied of the modification of the white fibre microstructure and resting state regional neural activity.

The main strengths of the current study are as follows: a) the cause (meditation) preceded the effect (brain changes); b) there was a dose (meditation)-response (brain changes) relationship and c) The results were consistent with well-established biologic knowledge.

There is one important limitation of DTI that is essential to understanding when evaluating colour heat maps. This limitation is the assumption that there is a homogeneous fibre structure within a pixel. Due to the relatively large size of the pixels in DTI data (2–3 mm), a pixel often contains axonal tracts with multiple orientations. The inclusion of tracts with different orientations causes the pixel to lose anisotropy. The fact that grey matter displays low anisotropy is not due to a lack of axonal fibres but because the fibre architecture is convoluted with respect to the pixel size. These low anisotropy areas should not be interpreted as low contents of axonal tracts. A second limitation is that, despite matching by relevant factors such as gender, age, education and ethnic group, residual confounding (confusion variables not assessed in this study) cannot be completely ruled out. Another problem is that the time factor (8 years of meditation) is long. It is possible that changes occurring during this period, which have nothing to do with the paradigm itself, could reflect other major variables (lifestyle, employment, medical conditions, etc). Finally, there are some caveats that influence metabolite measurements, such as magnetic field inhomogeneity and cerebrospinal fluid (CSF) contamination and artefacts. It is expected that modern 3T scanners with smaller voxel analysed will overcome these limitations. Pitfalls in MRS can be minimised using automated and standard protocols. Given that the spectral patterns are well known, minor artefacts are relatively easy to identify and read through. The 1H-MRS signal arises from grey and white matter and represents an ensemble average of multiple different cell types, including glial cells and neurons. However, these issues do not ameliorate the utility and reproducibility of this technique. The quantification of absolute metabolite values is complex, but the systematic errors might be lessened through comparisons with the control group and the use of the Creatine (Cr) ratios.

### Conclusions

mI, NAA and Glu have been identified as the most important altered metabolites in spectroscopic research in the brain of meditators. Further research should examine the thalamus, as the crossing point of several fronto-striato-thalamo-frontal brain circuits, for a better understanding of the neurochemical mechanisms implicated in the pathophysiology of meditators. The current study has confirmed the clinical role of diffusion-weighted imaging in long-term meditators. Statistically significant alterations of ADC in parietal white matter were correlated with years of meditation. The data obtained from the present study confirm that diffusion tensor imaging has a limited role in the evaluation of the cortical and white matter changes that occur in meditators.
